# Preimplantation Genetic Testing for a Chinese Family With X-Linked Lymphoproliferative Syndrome Type 1

**DOI:** 10.3389/fgene.2020.550507

**Published:** 2020-11-04

**Authors:** Songchang Chen, Weihui Shi, Yeqing Qian, Liya Wang, Junyu Zhang, Shuyuan Li, Yiyao Chen, Chunxin Chang, Hongjun Fei, Lanlan Zhang, Hefeng Huang, Chenming Xu

**Affiliations:** ^1^The International Peace Maternity and Child Health Hospital, School of Medicine, Shanghai Jiao Tong University, Shanghai, China; ^2^Shanghai Key Laboratory of Embryo Original Diseases, Shanghai, China; ^3^Key Laboratory of Reproductive Genetics (Zhejiang University), Ministry of Education, Hangzhou, China

**Keywords:** X-linked lymphoproliferative disease, preimplantation genetic testing, *SH2D1A* gene, nested PCR reaction, targeted next generation sequencing, haplotyping analysis

## Abstract

**Background:**

X-linked lymphoproliferative disease (XLP) is a rare primary immunodeficiency disorder. We performed experiments based on two strategies of preimplantation genetic testing (PGT) for a family with XLP caused by a mutation in *SH2D1A* (c.191G > A).

**Methods:**

First, a single-cell polymerase chain reaction (PCR) protocol was established using single lymphocytes. A nested PCR experiment was performed with direct sequencing after whole genome amplification of single cells to assess the accuracy of the genetic diagnosis. Embryos obtained after intracytoplasmic sperm injection were biopsied on day 3 and detected using the established single-cell PCR protocol. In the second PGT cycle, targeted next generation sequencing (NGS) was performed and the single nucleotide polymorphism (SNP) markers flanking *SH2D1A* were selected to determine the disease-carrying haplotype phase in each embryo.

**Result:**

In the first PGT cycle, six embryos were biopsied. Discounting an embryo from a single failed PCR experiment, five embryos were identified, including three unaffected and two hemizygous. After PCR, one normal embryo was transferred when it was developing into an early blastocyst. Although the ultrasound images indicated a viable singleton pregnancy, the implantation was on the cesarean scar. Therefore, an artificial abortion was performed. In the haplotyping cycle, six embryos were identified to have inherited a haplotype without pathogenic mutations. After the embryo implantation process failed twice, a successful singleton pregnancy was established, and subsequently, a healthy female child was born.

**Conclusion:**

Targeted NGS with haplotyping analysis circumvents the laborious process of multiplex PCR and is more likely to ensure diagnostic accuracy. However, when a genetic recombination occurs close to the site of mutation, confirmed identification using selected SNP markers can be challenging.

## Introduction

X-linked lymphoproliferative disease (XLP) is a rare life-threatening immunodeficiency disorder with an estimated incidence rate of 1–3 per million among males (Blackburn et al., 2019). XLP is characterized by a severely dysregulated immune response following infection by Epstein-Barr virus (EBV). Clinical manifestations vary from the establishment of an asymptomatic carrier state to a fatal or near-fatal infection. Typical symptoms in affected individuals include fulminant infectious mononucleosis, hemophagocytic lymphohistiocytosis, dysgammaglobulinemia, and lymphoma (Tangye, 2014). Approximately 40–60% of XLP cases, classified as XLP type 1 (XLP1), are caused by germline mutations in *SH2D1A*, whereas a second type of the disease, in which patients harbor mutations in *BIRC4*, is categorized as XLP type 2 (XLP2); in the latter, patients present with recurrent splenomegaly, chronic colitis, and typical XLP phenotypes (Pachlopnik Schmid et al., 2011).

*SH2D1A*, which is located in chromosome Xq25, encodes signaling lymphocytic activation molecule (SLAM)-associated protein (SAP), which is an 128-amino acid protein containing an Src homology 2 (SH2) domain (Tripathi et al., 2019). SAP has been reported to be expressed in T cells, natural killer (NK) cells, invariant natural killer T (iNKT) cells, eosinophils, and platelets, and acts as an essential adapter molecule that regulates signal transduction in T and NK cells by interacting with SLAM family immunomodulatory receptors (Ishimura et al., 2019).

Mutations in *SH2D1A* lead to the suppression/inhibition of SAP expression, or variable structural changes in SAP, which impairs SLAM-SAP interaction and downstream signaling, which may consequently induce T-cell overactivation and lead to defects in NKT cell ontogeny (Morra et al., 2001; Nichols et al., 2005). According to the “Databases for Immunodeficiency-causing variations”^1^ and the Human Gene Mutation Database (HGMD), to date, more than 130 *SH2D1A* mutations have been reported worldwide. However, there are no obvious correlations between the clinical manifestations of XLP and the types of *SH2D1A* mutations (Morra et al., 2001).

Couples carrying a pathogenic variant of *SH2D1A* have a 50% risk of transmitting the mutation in each pregnancy. As an early form of prenatal diagnosis, preimplantation genetic testing (PGT) offers a method for carriers or patients to prevent the transmission of genetic diseases by the transfer of unaffected embryos; the method can divided into three subtypes: testing for monogenic diseases (PGT-M), chromosome structural rearrangements (PGT-SR), and aneuploidies (PGT-A) (Kuliev and Rechitsky, 2017). In this study, we studied an XLP female carrier with two severely immunodeficient male children harboring a nonsense pathogenic variant of *SH2D1A*. We performed successful PGT procedures and careful pregnancy management to help the mother conceive a healthy child.

## Materials and Methods

### Patient Description

**FIGURE 1:**
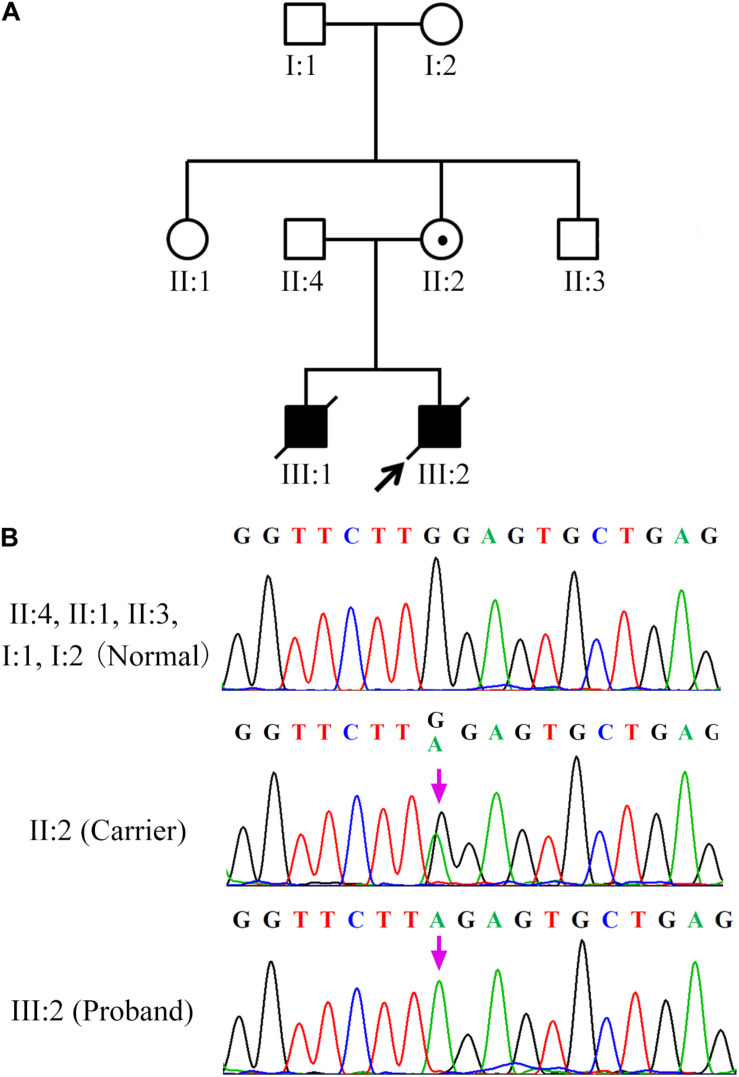
*SH2D1A* mutations in patients with X-linked lymphoproliferative disease **(A)** Affected members indicated in the pedigree chart for a Chinese family. The arrowhead denotes the proband. **(B)** Sequencing result of *SH2D1A* of specimens collected from the family members. The purple arrows indicate the mutation site at which the premature stop codon will be introduced.

A 32-year-old woman carrying an *SH2D1A* mutation was referred to our center for genetic consultation to prevent XLP inheritance in her offspring. The couple previously had two male children with severe EBV-associated hemophagocytic syndrome who died from overwhelming infections at 3 and 4 years, respectively. However, the couple did not have a family history of XLP. Blood samples were obtained from the family members, including the proband (III:2), his grandparents (I:1, I:2), parents (II:2, II:4), and the siblings of the mother (II:1, II:3) (Figure 1).

### Three-Dimensional Structure Prediction

The three-dimensional structures of wild and truncated SH2D1A were predicted using the Protein Homology/analogY Recognition Engine V 2.0 from the Structural Bioinformatics Group, Imperial College, London^2^.

### Single-Cell PCR

DNA extraction was performed according to the protocol described in the QIAamp DNA Blood Mini Kit (QIAGEN, Valencia, CA). Single lymphocytes from the peripheral blood samples of the couple were processed by whole genome amplification (WGA) using the SurePlex DNA Amplification System (BlueGenome, United Kingdom). Multiplex PCR was performed to co-amplify the *SH2D1A* and *SRY* loci. The primers used are listed in Table 1. The genotype of *SH2D1A* was determined by direct sequencing of the PCR products. After establishment of the single-cell PCR protocol, the technique was performed using specimens from the couple as the PGT procedure.

**TABLE 1:** Primers for the mutation region amplification of *SH201A* and *SRY.*

**Primer name**	**Forward primer (5′–3′)**	**Reverse primer (5′–3′)**	**Length (bp)**
SH2D1A-out	cctatgaatgcaatgacacca	aaacaggactgggaccaaaa	380
SH2D1A-in	ccattgttcttttggaatctttc	aacaattttggattggagcttt	250
SRY-out	gaatattcccgctctccgga	gctggtgctccattcttgag	470
SRY-in	acgggagaaaacagtaaaggcaac	ctgcaattcttcggcagcatcttc	287

### *In vitro* Fertilization and Blastomere Biopsy

This study was approved by the Medical Institutional Review Board of the International Peace Maternity and Child Health Hospital (IPMCH), Shanghai Jiao Tong University School of Medicine. The patient was treated with FSH (Gonal-F; Serono) after pituitary function was downregulated by treatment with a gonadotrophin-releasing hormone (GnRH) agonist (GnRH-a, Decapeptyl; Ferring). Follicular development was monitored by serial vaginal ultrasound imaging and measurement of serum E2 levels. Oocytes were retrieved at 36 h after human chorionic gonadotropin injection under ultrasound guidance and were subsequently fertilized by intracytoplasmic sperm injection (ICSI). The fertilized zygotes were cultured in G1 medium (Vitrolife, Sweden) at 37°C in a humidified atmosphere with 6% CO_2_. Single-cell biopsy was performed on day 3 after fertilization, and the DNA was amplified using the REPLI-g Single Cell Kit (Qiagen) according to the instructions. For each biopsied cell, a blank control was prepared from the final wash drop.

### Preimplantation Genetic Haplotyping (PGH)

Genomic DNA (gDNA) of the parents was extracted from their peripheral blood specimens and from the villus of the last fetus using the DNeasy Blood & Tissue Kit (Qiagen) according to the manufacturer’s instruction. The WGA products of the 10 blastomeres and the gDNA of the couple and villus were used to construct libraries with 200 bp insert sizes. The libraries were used for target capture with a 3.4M sequence capture array (BGI) containing the target regions. The post-capture libraries were sequenced by 90 bp paired-end sequencing using the Illumina Hiseq-2,000 (San Diego, CA, United States). The reads with low quality and adapter contamination were excluded by filtering before alignment. The cleaned reads were used to construct the haplotype phases of the parents and embryos. The haplotype phases of embryos from the parents were used to determine whether the embryos carried the pathogenic mutations.

## Results

### Preclinical Study

During the preclinical stage of PGT-M, *SH2D1A* isolated from the family members (III:2, II:4, II:2, II:1, II:3, I:1, I:2) was sequenced to identify the disease-causing mutations. Both the proband (III:2) and his mother (II:2) harbored a single base pair substitution c.191G > A (p.W64X) in *SH2D1A*, which was classified as a pathogenic mutation according to the American College of Medical Genetics and Genomics (ACMG) guideline (Richards et al., 2015). It was predicted to introduce a premature stop codon in the middle of exon 2, which would lead to the loss of 65 terminal amino acids from the C-terminal end of the protein. However, the *SH2D1A* variant was absent in his father (II:4) and grandparents (I:1, I:2), as well as in the siblings of his mother (II:1, II:3), which indicated that the *de novo* mutation originated from his mother (Figure 1).

### Optimization of Single-Cell PCR

**FIGURE 2:**
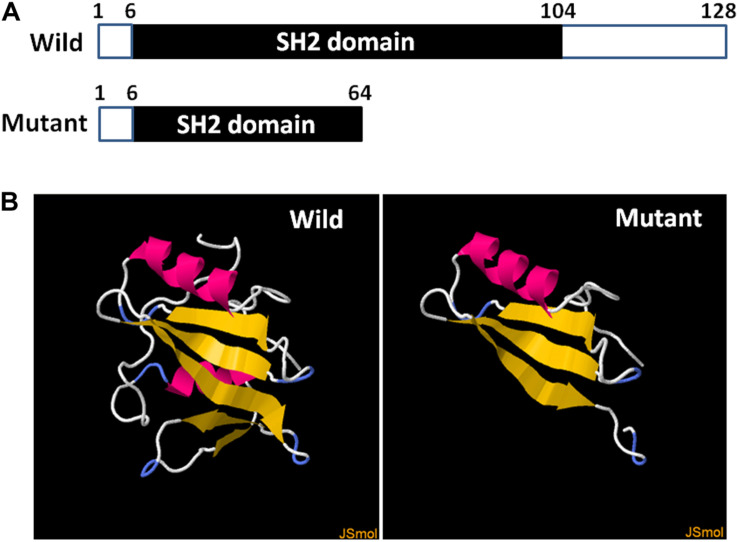
Diagrammatic representation and prediction of the three-dimensional structure of wild and truncated SH2D1A. **(A)** The numbers above the bars correspond to the amino acid number. **(B)** The yellow strands represent β-strands and the pink strands represent the α-helixes. The three-dimensional structure of the truncated protein was completely disrupted after *SH2D1A* mutation (c.191G > A).

Hundred single lymphocytes isolated from the specimens provided by the couple were analyzed. All the samples were suitable for PCR after WGA. The amplification rate of the mutation loci was 96.7%. Two out of a hundred single lymphocytes did not display the amplification signal, which could be attributed to the failure in the transfer of the single lymphocyte into the reaction tubes. Eight PCR amplifications were affected by allele drop-out (ADO); resultantly, the successful amplification rate of individual cells was 91.8%. None of the blank controls displayed amplification signals, which indicates that the results of single-cell PCR experiments were credible. The three-dimensional structure prediction revealed that SAP was composed of five β-strands and two α-helixes. However, the *SH2D1A* mutation (c.191G > A) led to the formation of a truncated protein with a disrupted structure (Figure 2).

### First PGT Cycle and Cesarean Scar Pregnancy

**FIGURE 3:**
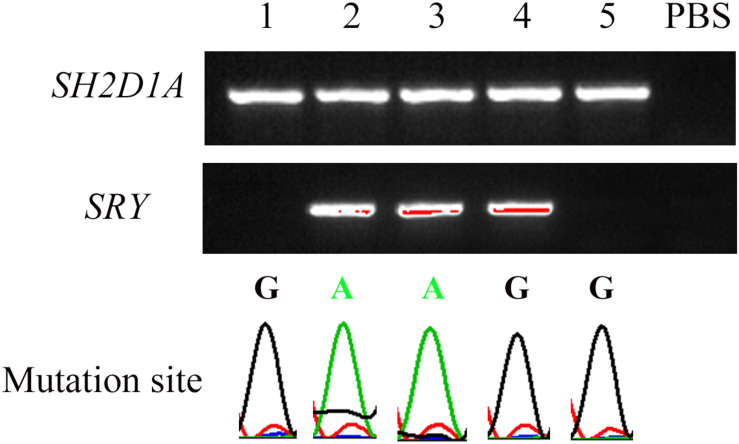
Nested PCR of five embryos after single-cell WGA. The peaks under the bands indicate the corresponding mutation site in *SH2D1A*. *SRY* was used to distinguish sex. “PBS” represents the negative control in which PBS was used as a PCR template.

Among the 10 embryos fertilized by ICSI, six were suitable for biopsy. DNA extracted from the blastomeres formed from each of the six embryos were amplified using WGA. Barring one embryo from a single failed PCR experiment, five embryos were identified, including three unaffected and two hemizygous (Figure 3). One normal embryo was implanted, and the 50-days ultrasound image showed the establishment of a viable singleton pregnancy. However, the embryo was implanted in the cesarean scar. Considering the serious complications of cesarean scar pregnancy (CSP), such as placenta previa, placenta accreta, uterine rupture, and catastrophic hemorrhage, an artificial abortion was performed. The other two normal embryos failed to implant after transplantation.

### PGT With Haplotyping Analysis

One year later, the couple re-attempted PGT using the haplotyping method, which uses suitable single nucleotide polymorphism (SNP) markers present in the flanking region of *SH2D1A*. We sequenced all the SNPs in the DNA samples of the couple and their son with a sequencing depth > 10 × within 1 Mb of *SH2D1A* and filtered the heterozygous SNPs in the X chromosome of the wife and the SNPs in the X chromosome of DNA isolated from the villus and the unaffected husband. We detected 3 SNPs that could be used to characterize the X chromosome of the father and 148 and 134 SNPs for the two X chromosomes of the mother. The SNP markers were selected to identify the disease-carrying haplotype phase in each embryo. Owing to the lack of adequate SNP markers, it could not be determined whether the four embryos harbored the disease-associated haplotype. Six embryos were observed to have inherited a haplotype without pathogenic mutations (Table 2). Unfortunately, the transferred embryos failed to implant in the first two processes. In the third transfer cycle, a successful singleton pregnancy was established with progesterone supplementation, and prenatal diagnosis via amniocentesis was in consonance with the PGT result. Eventually, the mother gave birth to a healthy female child with an Apgar score of 10 at 5 min after a cesarean section.

**TABLE 2:** Results of the haplotype-based linkage analysis.

**Sample ID**	**Gene**	**CHR**	**Gender**	**F(X)^a^**	**M(X1)^b^**	**M(X2)^c^**	**Haplotype**	**Results**
Embryo1	SH2D1A	chrX	F	0	0	0	-	-
Embryo2	SH2D1A	chrX	M	0	137	1	M(X1)	N
Embryo3	SH2D1A	chrX	F	0	0	0	-	-
Embryo4	SH2D1A	chrX	F	0	63	0	M(X1)	N
Embryo5	SH2D1A	chrX	F	1	95	0	M(X1)	N
Embryo6	SH2D1A	chrX	F	0	0	0	-	-
Embryo7	SH2D1A	chrX	F	2	96	1	M(X1)	N
Embryo8	SH2D1A	chrX	F	1	69	1	M(X1)	N
Embryo9	SH2D1A	chrX	F	0	0	0	-	-
Embryo10	SH2D1A	chrX	F	2	26	0	M(X1)	N

*CHR, chromosomes; F(X), X chromosome from father; M(X1), X chromosome without pathogenic mutations from mother; M(X2), X chromosome with pathogenic mutations from mother.*

*^*a*^Number of SNP markers in F(X).*

*^*b*^Number of SNP markers in M(X1).*

*^*c*^Number of SNP markers on M(X2); N, normal.*

## Discussion

X-linked lymphoproliferative disease (XLP) leads to a disorder involving the immune system and blood-forming cells and primarily occurs in males. Female carriers of XLP are always asymptomatic and remain undiagnosed until they have an affected son, similar to the mother who harbored a *de novo* pathogenic *SH2D1A* variant identified in our study. The pathogenic *SH2D1A* mutation had been reported in three unrelated families; affected males presented with fulminant infectious mononucleosis or lymphoproliferative disorders (Sumegi et al., 2000; Sandlund et al., 2013). If the pathogenic variant was not detected in the leukocyte DNA from the mother, a germline mosaicism, which has been reported in XLP (Schuster et al., 1993), could have been considered to explain the significantly similar disease manifestations in the two male children who died owing to immunodeficiencies following EBV infection. However, in certain rare cases, female carriers of XLP present with follicular lymphoma or hemophagocytic lymphohistiocytosis owing to skewed X-inactivation, which suggests that it is necessary for females with XLP kindreds to perform genetic testing and monitor typical symptoms of XLP (Woon et al., 2008; Holle et al., 2015). In this study, physical examination of the female carrier before the *in vitro* fertilization procedure and during her pregnancy revealed no obvious abnormalities, which indicates the immunological function of the mother was normal.

Various *SH2D1A* mutations, including deletions, insertions, nonsense, missense, and splice site mutations, have been identified (Sumegi et al., 2000). It was once considered that truncating mutations might be associated with more severe clinical presentations than missense mutations. Nevertheless, no significant genotype-phenotype correlation has been observed in XLP type 1 (Sumegi et al., 2002; Panchal et al., 2018). The clinical manifestations in patients differ even within a family, and may be associated with environmental factors (Filipovich et al., 2010).

PGT of embryos for the identification of monogenic disorders and chromosomal abnormalities aims to reduce the risk of the conception of an affected offspring by carriers or patients. Traditionally, ADO is one of the most important causes of the misdiagnoses of PGT owing to the preferential amplifications in PCR experiments (Stock-Myer and Johnson, 2018). In this study, two different strategies were adopted to address the problem after the WGA step. First, we used Sanger sequencing following ∼1,000-fold nested PCR to decrease the ADO rate, which was estimated to be maintained below 10%, as indicated by preclinical practices. In the second PGT cycle, targeted next generation sequencing (NGS) was performed to directly detect the mutation, and to select informative SNP markers for the haplotyping analysis. The haplotyping method based on the genotypes of the proband and the parents circumvents the laborious process of multiplex PCR, which is more likely to ensure diagnostic accuracy. Karyomapping is another linkage-based method for genetic diagnosis using genome-wide SNP genotyping that can be applied for multiple genes (Natesan et al., 2014). However, as a comprehensive method, karyomapping is relatively expensive. Moreover, haplotyping analysis may be challenging when a genetic recombination occurs close to the site of mutation owing to the fixed position of the SNP loci detected by karyomapping.

## Conclusion

In summary, we successfully performed the preimplantation genetic diagnosis for an XLP-affected family using two methods, nested PCR with direct sequencing and targeted NGS with haplotyping, both of which aimed to reduce ADO. Although the mother experienced a CSP and two failed embryo implantations initially, she eventually gave birth to a healthy female child. Our approach for PGT is applicable to other known monogenic diseases as well and indicates that careful supervision of pregnancy is indispensable for females with a history of cesarean section.

## Data Availability Statement

The datasets for this article are not publicly available due to concerns regarding participant/patient anonymity. Requests to access the datasets should be directed to the corresponding authors.

## Ethics Statement

The study was approved by the Ethics Committee of the International Peace Maternity and Child Health Hospital of Shanghai Jiao Tong University School of Medicine and the patients provided their written informed consent informed consent to participate in this study.

## Author Contributions

YQ, LW, HF, YC, SL, CC, and LZ carried out the experiments. JZ analyzed the data. SC made the figures. WS drafted the manuscript. CX and HH revised the manuscript. All authors approved the final version of the manuscript.

## Conflict of Interest

The authors declare that the research was conducted in the absence of any commercial or financial relationships that could be construed as a potential conflict of interest.

## Footnotes

1 http://structure.bmc.lu.se/idbase/SH2D1Abase/index.php

2 http://www.sbg.bio.ic.ac.uk/phyre2

## References

[B1] Blackburn P. R. Lin W. L. Miller D. A. Lorenzo-Betancor O. Edwards E. S. Zimmermann M. T. (2019). X-Linked Lymphoproliferative Syndrome Presenting as Adult-Onset Multi-Infarct Dementia. *J. Neuropathol. Exp. Neurol.* 78 460–466. 10.1093/jnen/nlz018 30990878 PMC6467195

[B2] Filipovich A. H. Zhang K. Snow A. L. Marsh R. A. (2010). X-linked lymphoproliferative syndromes: brothers or distant cousins? *Blood* 116 3398–3408. 10.1182/blood-2010-03-275909 20660790 PMC2981470

[B3] Holle J. R. Marsh R. A. Holdcroft A. M. Davies S. M. Wang L. Zhang K. (2015). Hemophagocytic lymphohistiocytosis in a female patient due to a heterozygous XIAP mutation and skewed X chromosome inactivation. *Pediatr. Blood Cancer* 62 1288–1290. 10.1002/pbc.25483 25801017

[B4] Ishimura M. Eguchi K. Shiraishi A. Sonoda M. Azuma Y. Yamamoto H. (2019). Systemic Epstein-Barr Virus-Positive T/NK Lymphoproliferative Diseases With SH2D1A/XIAP Hypomorphic Gene Variants. *Front. Pediatr.* 7:183. 10.3389/fped.2019.00183 31231620 PMC6558365

[B5] Kuliev A. Rechitsky S. (2017). Preimplantation genetic testing: current challenges and future prospects. *Expert Rev. Mol. Diagn.* 17 1071–1088. 10.1080/14737159.2017.1394186 29039978

[B6] Morra M. Simarro-Grande M. Martin M. Chen A. S. Lanyi A. Silander O. (2001). Characterization of SH2D1A missense mutations identified in X-linked lymphoproliferative disease patients. *J. Biol. Chem.* 276 36809–36816. 10.1074/jbc.M101305200 11477068

[B7] Natesan S. A. Bladon A. J. Coskun S. Qubbaj W. Prates R. Munne S. (2014). Genome-wide karyomapping accurately identifies the inheritance of single-gene defects in human preimplantation embryos in vitro. *Genet. Med.* 16 838–845. 10.1038/gim.2014.45 24810687 PMC4225458

[B8] Nichols K. E. Hom J. Gong S. Y. Ganguly A. Ma C. S. Cannons J. L. (2005). Regulation of NKT cell development by SAP, the protein defective in XLP. *Nat. Med.* 11 340–345. 10.1038/nm1189 15711562 PMC10655637

[B9] Pachlopnik Schmid J. Canioni D. Moshous D. Touzot F. Mahlaoui N. Hauck F. (2011). Clinical similarities and differences of patients with X-linked lymphoproliferative syndrome type 1 (XLP-1/SAP deficiency) versus type 2 (XLP-2/XIAP deficiency). *Blood* 117 1522–1529. 10.1182/blood-2010-07-298372 21119115

[B10] Panchal N. Booth C. Cannons J. L. Schwartzberg P. L. (2018). X-Linked Lymphoproliferative Disease Type 1: A Clinical and Molecular Perspective. *Front. Immunol.* 9:666. 10.3389/fimmu.2018.00666 29670631 PMC5893764

[B11] Richards S. Aziz N. Bale S. Bick D. Das S. Gastier-Foster J. (2015). Standards and guidelines for the interpretation of sequence variants: a joint consensus recommendation of the American College of Medical Genetics and Genomics and the Association for Molecular Pathology. *Genet. Med.* 17 405–424. 10.1038/gim.2015.30 25741868 PMC4544753

[B12] Sandlund J. T. Shurtleff S. A. Onciu M. Horwitz E. Leung W. Howard V. (2013). Frequent mutations in SH2D1A (XLP) in males presenting with high-grade mature B-cell neoplasms. *Pediatr. Blood Cancer* 60 E85–E87. 10.1002/pbc.24525 23589280 PMC4758190

[B13] Schuster V. Kress W. Friedrich W. Grimm T. Kreth H. W. (1993). X-linked lymphoproliferative disease. Detection of a paternally inherited mutation in a German family using haplotype analysis. *Am. J. Dis. Child* 147 1303–1305. 10.1001/archpedi.1993.02160360045015 8249949

[B14] Stock-Myer S. Johnson M. (2018). A big step forward for PGT-M? *Reprod. Biomed. Online* 37 126–127. 10.1016/j.rbmo.2018.06.017 30075839

[B15] Sumegi J. Huang D. Lanyi A. Davis J. D. Seemayer T. A. Maeda A. (2000). Correlation of mutations of the SH2D1A gene and epstein-barr virus infection with clinical phenotype and outcome in X-linked lymphoproliferative disease. *Blood* 96 3118–3125. 11049992

[B16] Sumegi J. Seemayer T. A. Huang D. Davis J. R. Morra M. Gross T. G. (2002). A spectrum of mutations in SH2D1A that causes X-linked lymphoproliferative disease and other Epstein-Barr virus-associated illnesses. *Leuk. Lymphoma* 43 1189–1201. 10.1080/10428190290026240 12152986

[B17] Tangye S. G. (2014). XLP: clinical features and molecular etiology due to mutations in SH2D1A encoding SAP. *J. Clin. Immunol.* 34 772–779. 10.1007/s10875-014-0083-7 25085526

[B18] Tripathi J. K. Sharma A. Gupta K. Abdelrahman H. Chauhan P. Mishra B. B. (2019). Function of SLAM-Associated Protein (SAP) in Acute Pneumoseptic Bacterial Infection. *J. Mol. Biol.* 431 4345–4353. 10.1016/j.jmb.2019.07.002 31295456 PMC11126331

[B19] Woon S. T. Ameratunga R. Croxson M. Taylor G. Neas K. Edkins E. (2008). Follicular lymphoma in a X-linked lymphoproliferative syndrome carrier female. *Scand. J. Immunol.* 68 153–158. 10.1111/j.1365-3083.2008.02128.x 18702745

